# Relative Validity of the Food Recording Smartphone App Libro in Young People Vulnerable to Eating Disorder: A Preliminary Cross-Over Study

**DOI:** 10.3390/nu17111823

**Published:** 2025-05-27

**Authors:** Melissa Basso, Liangzi Zhang, George M. Savva, Kathrin Cohen Kadosh, Maria H. Traka

**Affiliations:** 1 School of Psychology, Faculty of Health and Medical Sciences, University of Surrey, Guildford GU2 7XH, UK; melissa.basso@surrey.ac.uk (M.B.);; 2 School of Biosciences, Faculty of Health and Medical Sciences, University of Surrey, Guildford GU2 7XH, UK; 3 Food and Nutrition National Bioscience Research Infrastructure, Quadram Institute Bioscience, Norwich Research Park, Rosalind Franklin Rd., Norwich NR4 7UQ, UK; liangzi.zhang@quadram.ac.uk; 4 Core Science Resources, Quadram Institute Bioscience, Norwich Research Park, Rosalind Franklin Rd., Norwich NR4 7UQ, UK

**Keywords:** food recording, dietary intake, nutrition, young people, eating disorders, dietary assessment

## Abstract

**Background**: Dietary intake plays a crucial role in health research, yet existing methods for its measurement can lead to participant burden, lengthy recording, and human errors, and do not account for age-specific variations. Libro is a real-time diet-tracking mobile-based app offering flexible features. An automated food recording program within Libro was customized for young people vulnerable to eating misbehaviour. This preliminary study assessed its relative validity using a self-administered 24 h recall method as the reference method. **Methods**: The relative validity of Libro was tested by adopting a cross-over design that recorded food intake over a period of 3 non-consecutive weekdays and 1 weekend day with both methods. The participants were recruited online through a mental health research charity, and this study was conducted fully online. The primary outcome was the concordance of total energy intake between the two methods, with secondary outcomes focusing on the intake of protein, carbohydrates, fats, free sugars, fibre, and trans-fatty acids. Test–retest validity was assessed per method with the intraclass correlation coefficient; a Bland–Altman plot and *t*-test were performed to test agreement at the group level; correlation coefficient and cross-classification were performed to assess agreement at the individual level. **Results**: Forty-seven participants were included in the final analysis. The average intraclass correlation coefficient for energy intake measured by Libro over four days was 0.85 (95% CI: 0.76–0.91). Compared to Intake24, the average energy intake recorded using Libro was significantly lower (mean difference: −554 Kcal, 95% CI: −804.1 to −305.6 Kcal, *p* < 0.001), potentially driven by the reduced reporting of foods rich in free sugars. The correlation coefficient for average energy intake measured by Libro vs. Intake24 was 0.32 (95% CI: 0.03, 0.55), with only 27.7% of subjects classified in the same quartile with both methods (κ = 0.31, 95% CI: −0.03, 0.55). Concordance varied across specific dietary component measures. **Conclusions**: While Libro had good test–retest reliability if adopting a multiple administration method, it underreported energy and other aspects of dietary intake, along with poor classification performance compared to Intake24 in a population vulnerable to eating misbehaviour. We suggest that future studies improve user experience to increase compliance and data accuracy.

## 1. Introduction

Measurements of food choices and dietary intake are central to much health- and diet-related research. Current gold standard methods include recovery biomarkers, which are objective measures of nutrient status. However, they are expensive, do not capture the full complexity of dietary behaviour, and only exist for a subset of nutrients [1]. Self-reported dietary assessment methods are thus more commonly used and include food frequency questionnaires (FFQs), 24 h recalls (24HRs), and food recording (FR) diaries. FFQs measure dietary intake over a longer time, usually 6 to 12 months, categorizing similar food items and quantifying their frequency of consumption. In contrast, 24HRs assess the details of diet during the previous 24 h. This makes the instrument more precise in nutrient intake (NI) calculation, though more susceptible to day-to-day variation which can be accounted for by the collection of multiple, non-consecutive recalls. FR methods also assess diet over a shorter period, yet the recording process is carried out in real time, potentially correcting for retrospective memory bias, but subject to reactivity [2].

Despite the higher precision of 24HR and FR methods compared to FFQs, they still face several challenges such as (i) random recording errors; (ii) person-specific biases related to, e.g., motivation, age, and gender; (iii) high participant burden for data entry, cognitive load, portion size estimation, and memory; and (iv) social desirability of food and psychological vulnerability that could trigger underreporting or eating pattern alteration [3,4]. Adolescents and young people may face additional hurdles due to sporadic and social eating and heightened risk for the development of maladaptive eating behaviour [5,6]. While random errors can be mitigated through repeated measures, systematic errors consistently deviate from true values and may be method-specific, leading to biases in results [4]. To ensure good reliability and accuracy, new methods need to be validated against existing ones. When a direct comparison to unbiased gold standard methods is not feasible, validation studies should select a reference method that is sensitive to different types of errors and has previously established a certain degree of validity [7]. Accordingly, these studies measure relative as opposed to absolute validity, a factor that should be considered in results interpretation. It is also important that validation studies are conducted across different demographic groups acknowledging age-specific variations that could occur in energy demands, eating behaviour, and self-reporting accuracy [8,9].

Technology-based tools, particularly in a mobile format, are preferred by young people [10,11,12,13]. Digital tools ease dietary data collection through, e.g., multiple entry modalities, drop-down menus, and more automatised matching procedures to food databases [14]. Image-recognition methods are also available, although they are still in their infancy and are not acceptably precise [15]. At least two studies have validated digital FRs and compared them against 24HRs and found a poor performance for energy intake (EI), with mixed results for NI [16,17]. However, neither study accounted for age or demographic group as previously recommended. Also, both studies employed a 24 h interview as the reference method, conducted on the same day and pertaining to the same meals as the FR log. This experimental setup might bias the results by memory reinforcement processes and prompts of commonly forgettable food items during the interview, unlike the food log app evaluated. A recent meta-analysis further highlighted the challenges faced by dietary record apps by finding consistent underestimation of EI, with a pooled average discrepancy of −202 kcal/day compared to alternative methods, including 24HRs [7]. Macronutrient assessments also showed underestimations, with specific gaps for carbohydrates, fats, and protein. The review noted significant heterogeneity among studies, often due to varying study designs and population demographics. Importantly, studies using consistent food composition databases between the app and reference methods exhibited less variability, suggesting that methodological consistency could improve reliability. While digital FRs hold promise for reducing participant burden and increasing compliance, they may face challenges with prolonged recording periods, where adherence can decrease over time [10,16]. Refining these tools based on target group demographics and improving validation study designs could help address current limitations and ensure that dietary record apps can provide precise and reliable dietary assessments. Altogether, this underscores the need for tailored app development and robust validation practices to fully leverage the potential of these technological tools for accurate dietary assessment.

The aim of the present study is to test the adherence to and relative validity of a mobile-based FR app—the Libro app—against a web-based self-administered 24HR tool, i.e., Intake24, in a group of young people using a cross-over design. Given the heightened risk and prevalence of disordered eating behaviour in the target age group, we focused on populations known to be vulnerable, especially as vulnerability may not always be apparent and dietary assessment studies should carefully avoid potential triggers. Libro was chosen as it offers customizable programs and is equipped with a comprehensive food database [18,19]. We co-produced the program, choosing specific features based on feedback received from a consultation with young people. Intake24 utilizes the overlapping UK Nutrient Databank and was adopted as a reference method further because (i) it is based on a multiple-pass recall approach, consisting of a free recall followed by detailed questions—a system with accuracy comparable to face-to-face interviews [20]; (ii) its interface was developed for maximum engagement of adolescents and young people [21]; (iii) its relative validity was tested across various populations, including young people [21,22,23,24]; and (iv) it is sensitive to a different type of memory error: while Libro could be prone to prospective memory errors (forgetting to initiate a record), Intake24 relies on retrospective memory (recalling what was eaten).

## 2. Materials and Methods

### 2.1. Customization of the Libro Recording Program

The Libro app (Nutritics Ltd., Dublin, Ireland) is a mobile-based FR tool, requiring a basic level of literacy and familiarity with technology, designed for both consumer use and clinical or research applications. It is equipped with a comprehensive food database from different countries, including the official UK national databases, i.e., the 2021 McCance and Widdowson Composition of Foods Integrated Dataset and the Quadram labelling dataset [18,19]. It requires users to input detailed food entries for NI assessment, with features like barcode scanning and voice notes to assist data entry other than portion size examples. It presents customizable features that can be incorporated into an FR program, including trackers, physical activity recording, notifications, and media inclusion. A youth consultation group was conducted in March 2023 to refine the FR program design. Three young people (aged 23, 25, and 26 years), all with prior experience using an FR app—including two with a history of eating disorders—were recruited via the McPin Foundation, a UK-based mental health research charity. Eligibility criteria included being aged 14–27 and residing in the UK. The consultation was conducted remotely via Microsoft Teams, without camera or voice recordings, and facilitated by two researchers. Key recommendations included providing FR instructions in both written and video formats, incorporating frequent but customizable reminders, and offering examples for portion sizes to reduce burden. Participants highlighted the potential harm of trackers and recommended neutral phrasing for prompts. Virtual support was identified as a helpful addition to improving comfort and data quality.

Based on these insights, the FR program was customized as follows: instructions (written and video) were made accessible through the app, 4–5 daily notifications were included, and prompts addressed commonly forgotten ingredients (e.g., sugar and sauces). Trackers were disabled during this study, and prompts were neutrally phrased to minimize psychological risks. The researcher’s email was provided for support. The consultation also emphasized the importance of making the interface user-friendly, notifications customizable to individual schedules, and allowing for picture recognition—app-level changes that were not feasible. Details about the FR process and graphic interface can be found in Figures S1 and S2.

### 2.2. Validation of Intake24

Intake24 is an online multiple-pass 24HR method that utilizes the UK Nutrient Databank (using data from the McCance and Widdowson Composition of Foods Integrated Dataset). Across multiple studies, Intake24 has demonstrated good user-acceptability and validity as an online 24HR tool. In a comparison with interviewer-led multiple-pass recalls among 11–24-year-olds, Intake24 closely matched energy and macronutrient intakes—underestimating energy by just 1% on average (limits of agreement −49% to +93%) and yielding macronutrient and micronutrient means within 4% of the interviewer-led method for all nutrients except non-milk extrinsic sugars. The latter presents with age group differences, showing a ~3% underreporting in the 17–24-year-old subgroup [22]. Against the gold standard doubly labelled water measure of total energy expenditure in UK adults (40–65 years), Intake24 underreported EI by roughly 22–25%, with moderate correlations to total energy expenditure (r = 0.31–0.47). Test–retest reliability across recalls was modest for single administrations yet improved when averaging multiple days [23]. Finally, field testing in Scottish survey samples (aged 11+) found Intake24 to be user-friendly, engaging, and largely accurate in capturing dietary intake, with 44% of the users agreeing they would have used Intake24 more often, compared to 15% who disagreed [24].

### 2.3. Participants for the Validation Study

Participants were recruited through the McPin Foundation’s Young People’s Network, which consists of individuals aged 13–28 who are either interested in mental health research or have lived experience. This cohort can be considered at elevated risk for eating disorders for the following reasons: (i) adolescents and young adults are at highest risk for the onset of eating disorders [5]; (ii) the McPin network prioritizes the inclusion of individuals with lived experience of mental health issues, which is a population known to have increased vulnerability to eating disorders [25]; and (iii) online recruitment strategies through targeted mental health organizations tend to attract individuals with higher symptomatology or at greater risk for eating disorders [26]. Participants were considered eligible if aged 14–27 and currently living in the United Kingdom. No other eligibility criteria were applied other than computer/internet literacy. Each participant was asked to sign an online checkbox consent form before enrolment after reading a detailed study explanation and received a monetary reward at the study conclusion. Recruitment was conducted via mailout and delivery of this study was conducted remotely using Microsoft Teams. The sample size was determined based on the required precision in the correlation between the average measures obtained from the Libro and Intake24 methods. We expected a correlation coefficient of 0.8 as found in Mescoloto et al.’s (2017) study [27]. To achieve a 95% confidence interval width between 0.21 in Pearson correlation, based on the anticipated correlation of 0.8, it was necessary to enrol 50 subjects to participate in this study. A total of 49 participants were recruited. Ethical approval was received from the Ethics Committee of the University of Surrey (Reference number: FHMS 22-23 099). Given the relatively small sample size and gender imbalance (see Section 3.1), we define this study as preliminary in nature.

### 2.4. Experimental Design

**Figure 1:**
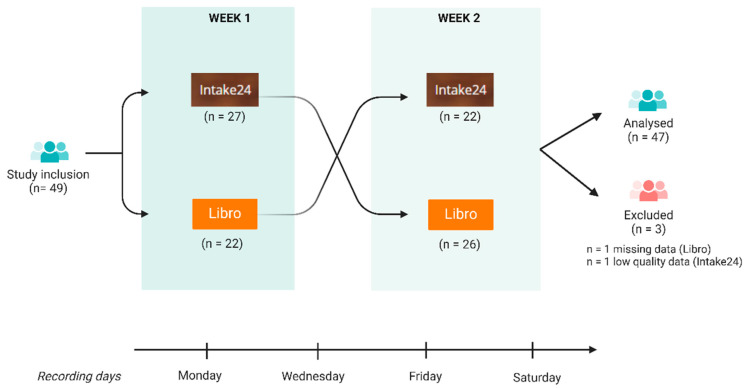
Cross-sectional experimental design and participant flow: Forty-nine participants were included in this study and assigned to each study branch by alternation. Twenty-two participants recorded their food intake using the Libro app on three alternative days (i.e., Monday, Wednesday, and Friday) and one weekend day (i.e., Saturday) during week 1. In the subsequent week (week 2), they followed the same recording protocol using Intake24. Twenty-seven participants first recorded their diet with Intake24, and subsequently with Libro. One participant failed to record their diet with Libro and was thus excluded from the analysis. One further participant was excluded due to implausible reporting of energy intake higher than 5000 Kcal with Intake24. Created with BioRender.com.

The dietary data were collected between May and August 2023, using the cross-over design illustrated in Figure 1. To obtain a mental health characterization of the sample, participants were asked to complete a set of validated self-report questionnaires online via the Qualtrics platform, following study inclusion. The administered measures included (i) the State–Trait Anxiety Inventory (STAI), which assesses trait anxiety (STAI-t) and state anxiety (STAI-s), and (ii) the Positive and Negative Affect Schedule (PANAS), which evaluates positive (PANAS-p) and negative (PANAS-n) affects. Score cut-offs for the STAI subscales are 20–37 (no/low anxiety), 38–44 (moderate anxiety), and 45–80 (high anxiety). Subsequently, scores for each PANAS subscale range from 10 (lowest) to 50 (highest). Subsequently, twenty-seven participants included in this study self-recorded their food intake using the Libro app (version 35) on three alternative weekdays (i.e., Monday, Wednesday, and Friday) and one weekend day (i.e., Saturday) during week 1. In the subsequent week (week 2), they followed the same self-recording protocol using Intake24. Conversely, 22 participants used Intake24 in week 1 followed by Libro in week 2. Participants were assigned to either sequence by alternation after study enrolment by the researcher leading the explanatory calls. The group size difference was due to a technical issue with the Libro app’s initialization, which meant that two participants initially allocated to the Libro–Intake24 sequence were reassigned to the alternate order.

### 2.5. Use of Libro and Intake24

An initial online appointment was arranged with each participant to deliver verbal instructions for both Libro and Intake24, without revealing which tool was being validated. Participants received an anonymised code and email address for free registration to Nutritics and log in to Libro. Reminder emails were sent before the first food log for both methods. A day before initiating the Libro program, participants were also notified via a welcoming message and an instructional video within the app. They were instructed to complete their food records in real time. In contrast, the Intake24 recordings followed a retrospective approach, where participants recorded their intake from memory after the day ended. The lead researcher monitored user activity in real time through the analytics platform of Nutritics and Intake24 and sent reminder emails for any missing food records.

### 2.6. Measures

To evaluate the validity of the Libro food record, dietary outcomes captured by Libro and Intake24 were compared. The primary outcome focused on total daily EI, while secondary outcomes were intakes of carbohydrates, fats, protein, fibres, free sugars (i.e., non-milk extrinsic sugars), and trans-fatty acids. The selection of carbohydrates, fats, and protein as secondary outcomes aligns with other nutritional studies [7], whereas fibres, trans-fatty acids, and free sugars were chosen based on their favourable (fibres only) and unfavourable effects on health and increasing research looking at diets high in such nutrients [28,29,30]. While micronutrients are important for overall health, they were not included in the present analysis. This decision was guided by this study’s focus on nutrients of emerging concern and the fact that most dietary validation studies primarily assess EI as the key benchmark [7]. Total EI can serve as a reasonable proxy for overall dietary quality, as Zhang et al. (2021) reported that smaller differences in EI between assessment methods were associated with smaller differences in micronutrient intake [7]. Further, micronutrients tend to have more gaps in food composition datasets. Both Intake24 and Libro have independently addressed these gaps using different methodologies, so differences in results between the two tools may partially reflect these methodological choices rather than differences in the foods reported.

### 2.7. Data Processing and Statistical Analysis

#### 2.7.1. Assessment of Adherence

Protocol adherence was quantified by the number of daily single recalls (SRs) completed, intended as a record of foods and drinks on a single given day, by subject, with each given method. Full adherence was intended as completion of all four assigned SRs; the difference in full adherence between methods was assessed with the McNemar Chi-square test and considered significant with *p*-value < 0.05. Subjects that did not complete any SRs with either Libro or Intake24 were excluded from the subsequent analysis.

#### 2.7.2. Data Quality Check and Data Cleaning

Data quality checks of SRs were carried out through data visualization with box plots at the end of data collection. Recorded portion sizes of outlying points were checked to explore potential recording errors. When possible, participants were also consulted when recording errors were suspected. Finally, we quantified underreporting within each method by calculating the percentage of SRs showing EI lower than 400 Kcal. To maximise data quality and minimise the impact of random recording errors, SRs reporting an EI higher than 5000 Kcal were excluded. Underreporting and overreporting thresholds were based on Willett’s exclusion recommendations [31] but adjusted to be less stringent due to the use of multiple recalls and the expected day-to-day variation in dietary intake. We compared results obtained with and without filtering these high values as a sensitivity analysis and no differences in trends and effects were detected. No filters were applied for SRs lower than 400 Kcal. Detailed results can be found in Figure S3.

### 2.8. Statistical Analysis

#### 2.8.1. Quantification of Single Recalls and Test–Retest Reliability

Day-to-day variation in total EI per participant was quantified as the difference between the highest and lowest single recalls and summarized using median and interquartile range (IQR) for each method, with a Wilcoxon test comparing methods. Test–retest reliability was assessed via intraclass correlation coefficients (ICCs) using a two-way mixed-effects model, estimating reliability for an average of four measurements. Correlation coefficients were calculated to distinguish method-related differences from normal daily variation.

#### 2.8.2. Assessment of Individual and Group Agreement Between Methods

We first calculated the mean average EI and NI across SRs for each participant using Libro and Intake24. A paired *t*-test and Bland–Altman plot assessed group-level agreement and differences in average intakes between methods. For the Bland–Altman plot, EI was represented by the median SRs, as mean values did not meet normality assumptions. A mixed-effects model was used to confirm differences in average EI between methods while controlling for order effects. Correlation coefficients (with 95% CI) evaluated individual-level agreement on a continuous scale, with a deattenuated coefficient computed following Trafimow et al. [32]. Cross-classification analysis, based on quartiles, assessed individual-level agreement on a categorical scale, with favourable outcomes defined as >50% in the same category and <10% in opposite categories. Kappa statistics (with 95% CI) quantified inter-rater reliability.

The choice of statistical tests followed Lombard and colleagues [33]. Normality was assessed using qq plots. Parametric tests and mean values were used for normal data; non-parametric alternatives and medians were used otherwise. A *p*-value of 0.05 was set as the threshold for significance. Average NI was summarized as energy density for comparison with National Diet and Nutrition Survey (NDNS) reference values. For analysis, NI was adjusted using the residual model with absolute NI as the dependent variable and total EI as the independent variable [34]. Five values (for free sugars and trans-fatty acids) slightly below zero after adjustment were capped to 0 for interpretability. Data processing and analysis were performed in R (version 4.3.0) using RStudio (version 2022.12.0) and the packages “networkD3 0.4”, “car 3.1-2”, “lme4 1.1-35.1”, and “irr_0.84.1” [35,36,37,38,39]. The anonymised data collected and the pre-processing and analysis scripts are available on GitHub, https://github.com/BMelissa/FRvalidation_against24HRs [40].

## 3. Results

### 3.1. Participants

Of the 49 participants included in this study, 1 did not complete any SRs in Libro, and 1 was excluded due to poor data quality, resulting in 47 participants for analysis. Fourteen participants were female (29.8%), the mean age (±standard deviation) was 23.2 (±2.4), and the mean BMI was 24.3 (±7.4) (see Table 1). Mean STAI-t and STAI-s were 43.1 (±9.6) and 40.5 (±11.8), respectively, indicating moderate-to-high levels of trait and state anxiety. The PANAS results indicated a positive affect score of 34.9 (±8.8) and a negative affect score of 22.7 (±7.1). Five participants had past or current diagnoses of a psychiatric disorder (two participants had eating disorders, and three had anxiety and/or depression).

### 3.2. Adherence

Of the 49 participants included in this study, 46 participants (94%) completed four SRs with Intake24, and the remaining 3 (6%) completed three SRs. Forty-three participants (88%) completed four SRs with Libro, 4 participants (8%) completed three SRs, 1 participant completed two SRs, and 1 completed none. Differences in full adherence did not differ significantly between the two methods (McNemar Chi-square test, McNemar’s Chi-squared value = 0.57, *p* = 0.45).

### 3.3. Results on Energy Intake

#### Single Recalls and Test–Retest Reliability

**Figure 2:**
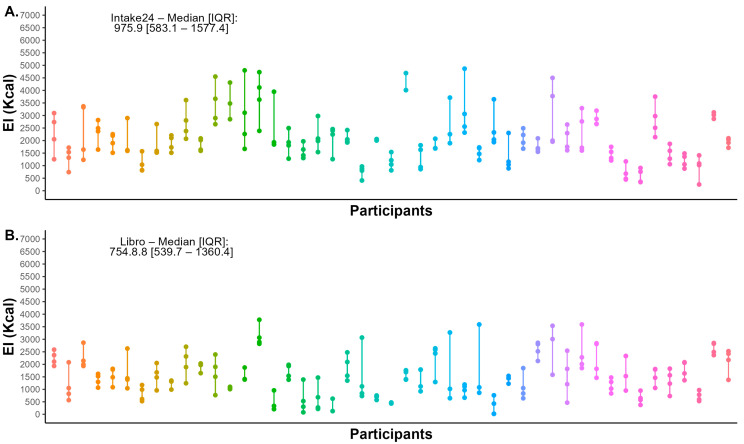
Day-to-day variations in energy intake per method. Day-to-day variations in the energy intake for each of the included 47 participants were captured using Intake24 (**A**) and Libro (**B**). Different colours refer to different participants. EI: energy intake; IQR: interquartile range.

Day-to-day variations in SRs per participant can be seen in Figure 2. The median of EI variation was 975.9 Kcal (IQR = 583.1–1577.4 Kcal) for Intake24 (in Figure 2A) and 754.8 Kcal (IQR = 539.7–1360.4 Kcal) for Libro (in Figure 2B) (paired sign-rank Wilcoxon test = 657, *p* = 0.33). The ICC was similar between methods: the ICC for the average over four days for Intake24 was estimated as 0.83 (95% CI: 0.73–0.90); the ICC for Libro was 0.85 (95% CI: 0.76–0.91). The correlation between single entries was moderate within both methods. For Intake24, the average Spearman correlation between pairs was 0.60 with a range of (0.55 to 0.71), and 95% confidence intervals spanning from 0.31 to 0.83. For Libro, the average Spearman correlation was 0.59 with a range of (0.48 to 0.64) and 95% confidence intervals from 0.23 to 0.78. These intervals reflect the variability in correlations and suggest moderate consistency across days within each method.

### 3.4. Individual and Group Agreement Between Methods

**Figure 3:**
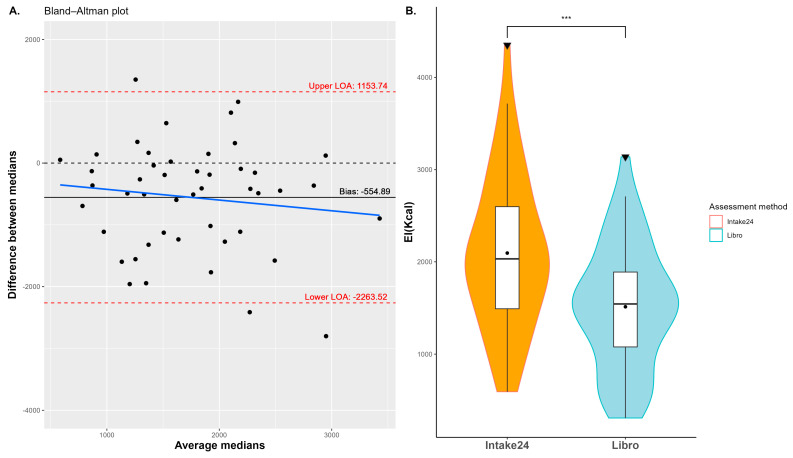
Group-level analysis between methods for 47 participants. (**A**) Bland–Altman plot: The differences between the medians of SRs are plotted against the averages of the medians, with limits of agreement indicated by the dashed red lines and bias by the continuous black line. The blue line is used to investigate if any trend in the differences across the range of measurements exists. (**B**) Violin and box plots: Violin and box plots of average energy intake (EI) in Kcal per participant as recorded by Intake24 (orange) and Libro (light blue). Triangles represent outliers. ***: *p*-value < 0.001.

The mean average daily EI across participants captured by Libro was 1512 Kcal, the median was 1542 Kcal, and the minimum and maximum values were 306 and 3138 Kcal. The average EI captured by Intake24 was 2094 Kcal, the median was 2031 Kcal, and the minimum and maximum values were 592 and 4351 Kcal. Qq plots for EI analysis can be seen in Figures S4–S6. The Bland–Altman plot estimated a bias of −554.9 Kcal (95% CI: −804.1 to −305.6 Kcal) with 95% limits of agreement being −2263.5 to 1153.7 Kcal (see Figure 3A). Visual inspection of the Bland–Altman plot shows no dependency of differences on average EI values. Agreement at the group level was also assessed using a paired *t*-test, which revealed a significant difference in EI assessment (t = 4.36, *p* < 0.001) as shown in Figure 3B. Analysis using linear mixed models confirmed a significant impact of the assessment method on EI variation (β= −586.84, *df* = 45, t = −4.56, *p* < 0.001), indicating that the method significantly influenced EI outcomes. In contrast, the sequence of method application did not have a significant impact (β= 188.46, *df* = 45, t = 1.48, *p* = 0.147).

**Figure 4:**
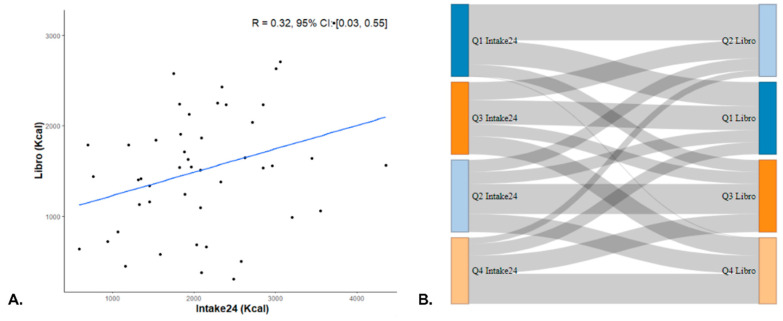
Individual-level analysis between methods for 47 participants. (**A**) Scatter plot of average individual energy intake (EI) values in Libro versus Intake24 with a linear regression line. (**B**) Sankey diagram depicting participant flow from one quartile as assessed by Intake24 (left) to the same or different quartile as assessed by Libro (right).

The Pearson correlation coefficient between average Intake24 and average Libro EI was 0.32 (95% CI: 0.03, 0.55) as illustrated in Figure 4A. After adjusting for the attenuation effect, the correlation coefficient rose to 0.38. Utilising a cross-classification approach as depicted in Figure 4B, 27.7% of the subjects were categorised in the same quartile, and 4.3% were placed in opposite categories. To further evaluate inter-rater reliability on a categorical scale, weighted Kappa statistics were employed, which indicated a fair agreement (κ = 0.31, 95% CI: −0.03, 0.55).

### 3.5. Results on Nutrient Intake

Summary statistics (mean and median intake) of NI can be found in Table 2, along with standard scores reported by the NDNS for the 19–64 age group. The NI is expressed in nutrient density (%) for visual comparability. The percentage of energy derived from fats, carbohydrates, and protein intake was comparable to the NDNS data for both Libro and Intake24. Free sugar intake was comparable for Intake24 only, whereas trans-fatty acid intake was comparable to NDNS data for Libro only.

We then applied individual- and group-level analyses to energy-adjusted NI (Table 3). Given the non-normal distribution of NI data, non-parametric methods were employed. Spearman correlation coefficients between methods ranged from −0.04 for fats to 0.27 for protein (all r values ≤ 0.27, see Figure S7). The confidence intervals for most nutrients included zero, indicating considerable variability and generally weak correlations, with the highest upper bounds observed for protein and free sugars. Assessment bias was quantified as median differences and ranged from −0.4 g (for trans-fatty acids) to −93.9 g (for carbohydrates), and the Wilcoxon signed rank test showed a p value lower than 0.05 for all analysed nutrients, suggesting that Libro significantly underestimated NI compared to Intake24. The biggest difference was seen for free sugars and trans-fatty acids, with 67% and 47% underreporting when using Libro, respectively. Cross-classification based on quartiles showed that the percentage of subjects that were correctly classified ranged between 12.8% for fats and 38.3% for protein and trans-fatty acids. Between 8.5% (for protein, fibres, free sugars, and trans-fatty acids) and 12.8% (for carbohydrates) of the participants fall within opposite categories. Weighted Kappa statistics varied from −0.09 for fats and 0.29 for protein with 95% confidence intervals extending from −0.35 to 0.56.

## 4. Discussion

The present preliminary study customized and validated a food recording program in the Libro app against the 24 h recall system Intake24 in a moderately anxious sample of young people vulnerable to eating misbehaviour. Both methods demonstrated comparable day-to-day variation and good test–retest reliability. However, Libro showed a poor correlation with Intake24, even with four measurements, underestimating EI by 27% (554 Kcal) with wide limits of agreement (−1153.7 to 2263.5 Kcal). This underreporting bias aligns with prior studies validating FR apps, which often find underestimated EI and mixed accuracy for nutrients [7]. For example, Ocke et al. [17] reported significant underestimation of certain nutrients, including carbohydrates and sugars, but not protein or sodium. Other authors used a controlled meal and found that participants significantly underreported their EI when using an FR app with wide limits of agreement. The same trend was seen with a 24HR method, albeit not significant. Worth noting, the FR app was generally described as easy to use and likely to be used daily [16]. Libro underreported free sugars by 67% and trans-fatty acids by 47% compared to Intake24. Few validation studies have concentrated on these nutrients, despite their significant health implications. Recognizing this gap, we included these nutrients in our assessment, relying on Intake24 being shown to report free sugars within 3% of interviewer-led recalls [21]. Our findings suggest Libro lacks accuracy for these nutrients, although future studies should perform reliability comparisons based on the number of single recalls. Given the high day-to-day variation in such nutrients, a higher number of single recalls might indeed be needed to capture true intake.

Prior work suggests that the underreporting bias observed with the Libro app might be attributable to the absence of memory prompts for commonly forgotten ingredients, a feature that Ocke and colleagues recommend including in self-reported dietary assessment tools [17]. The absence of tailored pop-up prompts in Libro, unlike Intake24, could also explain the relative underreporting of free sugars by increasing the omission of sugar-rich items such as table sugar, sauces, sweetened beverages, and snacks for recording. A comparison of nutrient density between Libro and Intake24 against NDNS data showed visually comparable values for protein, fats, carbohydrates, and trans-fatty acids. This might suggest that Libro might effectively gauge the proportion of NI, despite the general tendency to underestimate overall intake. However, the reported consumption of free sugars in Libro was still notably lower compared to the NDNS data, highlighting the need for further refinement in Libro’s methodology to enhance the accuracy of free sugar intake estimates.

Agreement at the individual level was poor, with only 27.7% of subjects accurately ranked by EI quartiles and 4.3% placed in opposite categories, although this could reflect genuine intake variation. Correlation and Kappa coefficients (~0.30) indicated fair agreement, with similar trends for NI estimation and high measurement error. Correlation coefficients for single entries within methods ranged from 0.48 to 0.71, consistent with the high ICC for averages. These results suggest that while Libro may be consistent within its own measures and present good test–retest reliability with multiple entries, it may not accurately capture individual dietary behaviours compared to Intake24.

Self-reported dietary assessment methods rely on participant compliance, motivation, and user experience, which are influenced by user-centric design. While we implemented several features discussed during co-production (e.g., media, instructions, notifications, and support), limitations in customizing the user interface and notification system might have contributed to lower engagement and underreporting in Libro. Participants, including young people, seem to prioritize the user interface layout and are more motivated by colourful and brighter icons [41,42]. An easy-to-use interface with a minimal number of layers is also preferred [43]. The youth consultation confirmed the importance of an intuitive, visually appealing interface, and notifications aligned with individual eating schedules. It is worth noting that notifications could be counterproductive if they do not coincide with a participant’s individual eating schedule, potentially disrupting the recording accuracy. We also hypothesize that the Libro app’s slower processing speed might have reduced accuracy and further suggest that gamification strategies (e.g., points, badges) with intrinsic motivators (e.g., competence, belonging), could further improve engagement and long-term compliance in future iterations [44]. Such software and engagement system improvements could be further supported by the inclusion of pictures of meals and free text descriptions. Leveraging current AI-based technology could then lead to a quicker and more accurate dietary intake assessment, although AI techniques are still under refinement [15,45].

The included sample was characterized by moderate-to-high anxiety levels. Alongside fear of negative evaluation, dieting history, and social desirability, anxiety has been associated with an increased risk of underreporting [46,47]. Notably, Tooze et al. (2004) identified distinct psychosocial predictors of underreporting depending on the dietary assessment method used [47]. This finding underlines the importance of conducting population-specific studies and measuring psychosocial factors when evaluating dietary intake. As this study was limited to FFQ and 24HR methods, more research is needed to identify FR-specific predictors of under- or overreporting. Such insights could enable nutrition trials to tailor dietary assessment tools to the characteristics of the target population, ultimately improving the accuracy of intake measurement.

Finally, it is crucial to consider that different types of eating disorders could influence dietary reporting in distinct ways, with anorexia patients tending to overreport, and obese individuals tending to underreport EI [48]. Such a reporting bias could affect the interpretability of nutritional studies, as measured intake might not accurately reflect real intake in a population-dependent manner. These considerations further corroborate the need for population-specific designs, participants’ stratification, or analysis adjustment for key traits. In validation studies, incorporating a psychological screening prior to the administration of the dietary assessment could also be advisable for a complete sample characterization that would support a more informed data interpretation.

### Strengths and Limitations

This study presents several strengths. First, by averaging multiple 24 h recalls per person, we minimized intra-individual variability due to day-to-day fluctuations in intake. Second, the chosen reference method was developed for maximum engagement of adolescents and young people specifically, with its validity tested within the age range of interest [21,22,23,24]. This makes Intake24 a reliable and strong benchmark for assessing Libro validity. Third, this study was population-specific and provided a clear psychological characterization of the included sample, thus offering detailed insights within a specific psychological framework. Finally, the integration of multiple statistical models allowed us to assess Libro from multiple perspectives, including its test–retest reliability and disentangling between individual and group assessment.

Despite the previous points, this study has several limitations. First, we did not collect data on participants’ experience with FR, which may have influenced data quality. Second, the sample was predominantly male (70%), as recruitment of additional female participants proved challenging despite extended efforts. Given the higher prevalence of eating disorders among females, future studies should aim for more gender-balanced samples and, where possible, conduct gender-specific analyses to explore potential differences in dietary reporting. Third, the online nature of this study could have impacted motivation, as monetary incentives were used without intrinsic reward strategies to improve compliance, particularly for higher-burden tasks like using the Libro app. Fourth, Intake24 recorded more entries with EI exceeding 5000 Kcal, likely due to portion size errors. We cannot rule out overreporting, although this is unlikely given Intake24’s validation in prior studies, the use of a multiple-recall protocol, and data filtering processes. Both tools use McCance and Widdowson’s Composition of Foods Integrated Dataset, but the lack of standardized methods for estimating nutrients like free sugars and trans-fatty acids, as well as distinct computational approaches, may have influenced results [49]. Also, despite concerns about week-to-week variability in dietary intake due to different collection weeks, our analysis suggests that differences derive from methodological differences between the tools. Finally, although we adopt a co-production approach for the customization of the program, future studies should consider incorporating a focus group pre- and post-testing and employing qualitative analysis tools such as NVivo to enhance data interpretation [50].

## 5. Conclusions

To the best of our knowledge, we conducted the first population-specific validation study, accounting for the psychological profile of the included participants—a factor known to influence dietary recording, potentially in a method-specific manner. Our preliminary study found that Libro demonstrated good test–retest reliability, yet it exhibited suboptimal performance in assessing dietary intake in a moderate-to-high anxious population vulnerable to eating misbehaviour. Such limitations might stem from the absence of tailored prompts for commonly forgotten items, insufficiently personalized notification systems, and a lack of integrated motivational strategies to sustain user engagement. Our findings underscore the importance of an integrated approach that considers both method-specific functionality and the unique characteristics and needs of the target population. Notably, method-related differences in how psychosocial factors influence underreporting may have contributed to our findings and warrant further investigation. Future research should prioritize advancing the app’s interface, incorporating adaptive technologies, and addressing user motivation to enhance compliance and the accuracy of dietary assessments. Integrating real-time, nutritionist-led guidance into Libro recordings could uncover additional areas for refinement. Such enhancements could establish Libro as a more reliable tool for dietary research and inform advancements in other FR apps by highlighting the importance of user-centric design, adaptive features, and the psychosocial context of the application.

## Figures and Tables

**Table 1:** Demographics and psychological profile of the included sample.

	Final Sample	Males	Females
	n = 47	n = 33 (70.2%)	n = 14 (29.8%)
Mean age (mean)	23.2 (±2.4)	23.6 (±1.7)	22.8 (±2.8)
Age range (mean)	19.1–27.1	20–27	19.1–27.1
Weight (Kg)	63 (±9.6)	65.2 (±7.9)	57.6 (±11.4)
Height (cm)	163.65 (±17.5)	164.6 (±19.8)	161.3 (±10.2)
BMI (Kg/m^2^)	24.3 (±7.4)	25.2 (±8.3)	22.2 (±4.4)
STAI-t (mean)	43.1 (±9.6)	42.7 (±8.2)	44.0 (±12.5)
STAI-s (mean)	40.5 (±11.8)	40.1 (±10.5)	41.2 (±14.8)
PANAS-p (mean)	34.9 (±8.8)	36.1 (±9.1)	32.0 (±7.8)
PANAS-n (mean)	22.7 (±7.1)	22.7 (±6.9)	22.5 (±7.7)

Data are mean ± standard deviation.

**Table 2:** Summary statistics per method and reference values with 47 participants included. Summary statistics of energy intake (expressed in Kcal) and nutrient intake (expressed in nutrient density) as assessed by Intake24 and Libro, and reference values from the National Diet and Nutrition Survey (NDNS) for ages 19–64.

	Intake24 Mean (SD)	Median (Lower-Upper 2.5 Percentile)	Libro Mean (SD)	Median (Lower-Upper 2.5 Percentile)	NDNS Mean (SD)	Median (Lower-Upper 2.5 Percentile)
Energy (Kcal)	2094 (828)	2031 (704–3691)	1512 (673)	1542 (386–2698)	1882 (628)	1815 (864–3176)
Protein (%)	17.1 (4.0)	17.0 (9.7–24.9)	20.8 (7.9)	19.4 (11.4–41.9)	16.5 (4.2)	16.0 (10.3–25.6)
Fat (%)	31.9 (5.5)	33.1 (22.5–39.9)	34.0 (6.4)	33.8 (24.4–47.1)	32.9 (6.6)	33.4 (18.9–44.9)
Carbohydrate (%)	49.8 (7.5)	49.5 (37.5–66.4)	43.3 (10.7)	45.8 (11.6–58.8)	45.5 (7.7)	45.5 (30.0–60.5)
Fibre (%)	1.5 (0.5)	1.4 (1.0–2.9)	2.3 (0.9)	2.3 (0.9–3.9)	-	-
Free sugars (%)	11.8 (5.8)	11.2 (4.6–22.2)	5.4 (4.1)	4.3 (0.0–14.6)	11.6 (6.2)	10.7 (2.4–25.0)
Trans-fatty acids (%)	0.4 (0.1)	0.4 (0.2–0.6)	0.7 (0.5)	0.5 (0.1–2.2)	0.7 (0.3)	0.6 (0.2–1.3)

SD: standard deviation.

**Table 3:** Statistical analysis of nutrient intake for 47 participants. Individual- and group-level analyses of energy-adjusted nutrient intake between Intake24 and Libro.

	Intake24 Median (IQR)	Libro Median (IQR)	Median Difference (95% CI)	Wilcoxon Signed Rank (*p*-Value)	Spearman (95% CI)	Cross-Classification % Same Quartiles	Cross-Classification % Opposite Quartiles	Weighted Kappa Stat (95% CI)
Protein (g)	90.2 (76.0–99.8)	67.5 (59.9–82.6)	−17.9 (−23.4–13.9)	<0.001	0.27 (−0.02–0.51)	38.30	8.5	0.29 (−0.01, 0.56)
Fat (g)	76.3 (67.8–81.5)	56.5 (51.9–61.7)	−20.6 (−26.8–13.8)	<0.001	−0.04 (−0.33–0.25)	12.77	10.6	−0.09 (−0.35, 0.17)
Carbohydrate (g)	273.7 (253.7–293.8)	179.8 (162.9–192.1)	−93.9 (−107.9–83.6)	<0.001	0.21 (−0.08–0.47)	34.04	12.8	0.19 (−0.16, 0.51)
Fibre (g)	14.3 (12.5–16.2)	16.6 (13.3–20.2)	2.1 (0.5–3.5)	0.009	0.20 (−0.1–0.46)	34.04	8.5	0.22 (−0.08, 0.52)
Free sugars (g)	61.7 (42.2–79.9)	20.3 (10.8–30.4)	−41.4 (−49.9–32.1)	<0.001	0.21 (−0.08–0.47)	31.91	8.5	0.19 (−0.08, 0.47)
Trans-fatty acids (g)	0.9 (0.8–1.0)	0.4 (0.3–0.6)	−0.4 (−0.6–0.3)	<0.001	0.16 (−0.13–0.43)	38.3	8.5	0.19 (−0.11, 0.46)

## Data Availability

All data are available at https://github.com/BMelissa/FRvalidation_against24HRs.

## Supplementary Materials

The following supporting information can be downloaded at https://www.mdpi.com/article/10.3390/nu17111823/s1. Figures S1–S7: Customization of the FR program in the Libro app (S1), Example of number of layers and food recording process. (S2), Daily single recalls of total Energy intake per each method (S3), Qq plots for median EI (S4), Qq plots for mean EI (S5), Qq plots for Libro–Intake24 differences (S6), Scatter plot of nutrient intake (S7).

## Abbreviations

The following abbreviations are used in this manuscript:

24HRs	24 h recalls
EI	Energy intake
FFQ	Food frequency questionnaire
FR	Food recording
NDNS	National Diet and Nutrition Survey
NI	Nutrient intake
