# Supplementary material for: Relative Validity of the Food Recording Smartphone App Libro in Young People Vulnerable to Eating Disorder: A Preliminary Cross-Over Study
Source: Nutrients. 2025 May 27;17(11):1823. doi: 10.3390/nu17111823 (PMC12157823; doi:10.3390/nu17111823)
Supplement: Supplementary file 1 [file nutrients-17-01823-s001.zip › nutrients-3642116-supplementary.pdf]

Supplementary materials

Customization of the Libro recording program

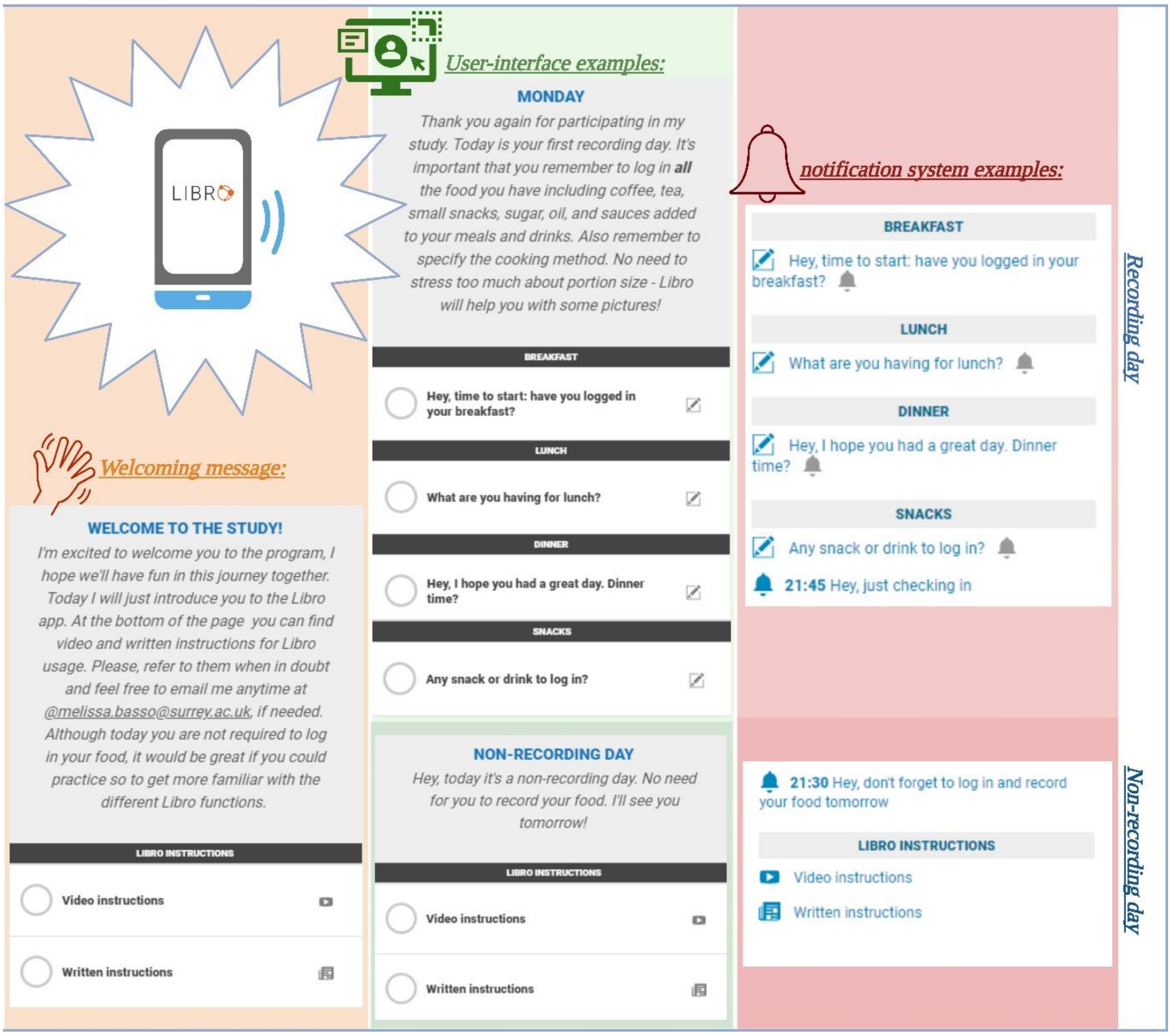

Figure S1. Customization of the FR program in the Libro app. Orange: the welcome message displayed before the recording week includes written and video instructions. These instructions remained accessible throughout the program. **Green:** User-interface (**top**) during a recording day, (**bottom**) during a non-recording day. Meals are displayed as a task which had to be ticked off for compliance checks via the Nutritics platform. **Red**: Example of the 4-5 push reminders during recording days (**top**) and **1**during non-recording days (**bottom**).

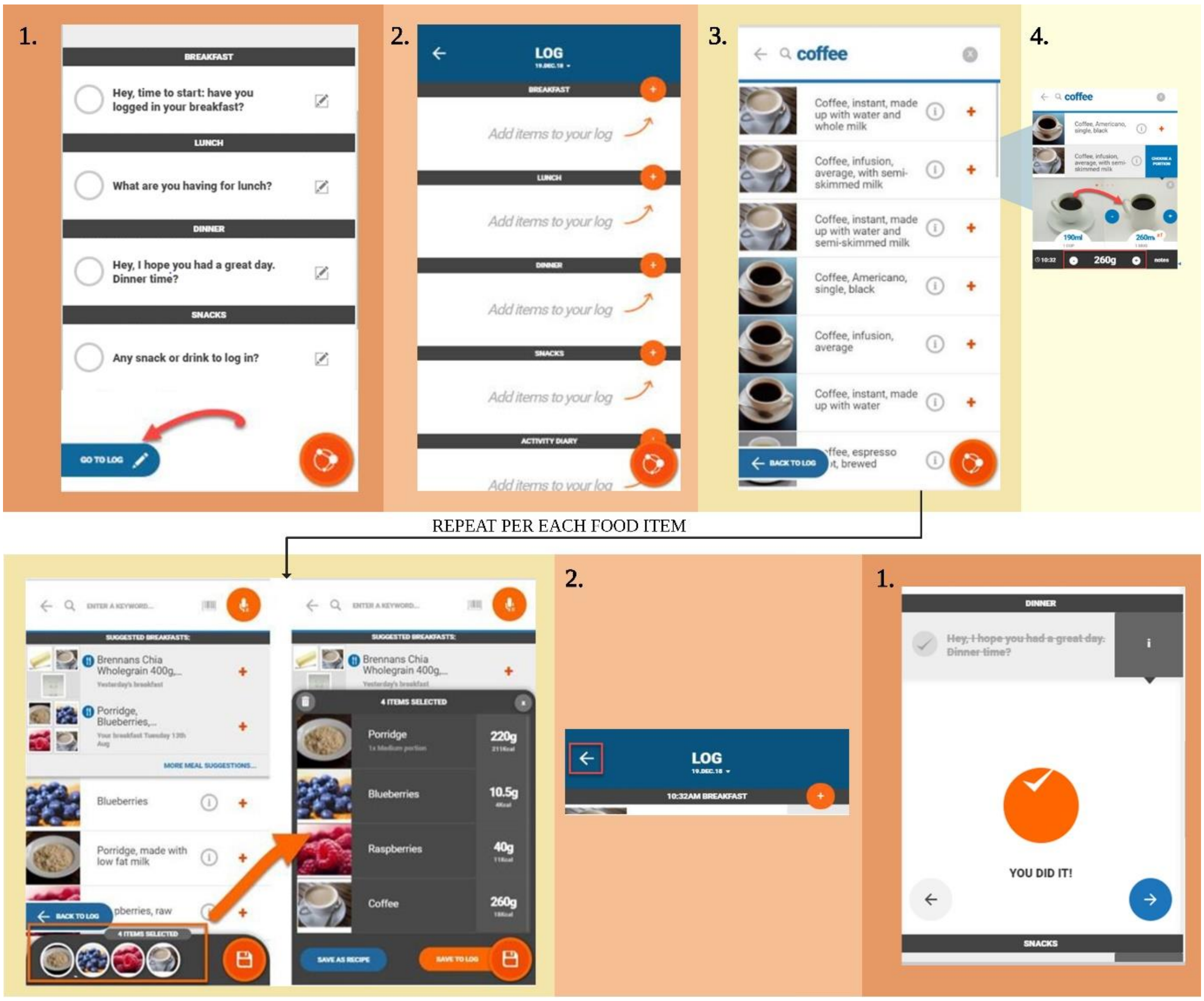

Figure S2. Example of number of layers and food recording process. Orange: layer 1; recording tasks are displayed, to start recording food participants are required to press the “go to log” button. **Light orange**: layer two; food log interface, to log food participants are required to press “+”. **Yellow**: layer 3; within each meal, participants can type food and select among options within a drop-down menu. Additional entry modalities were made possible (barcode, voice notes). **Light yellow**: layer 4; portion size selection, for some food items examples of portion size are shown. The same selection process is repeated per each food item consumed; all items are displayed in the grey window at the bottom and need to be saved before leaving the layer. Back to the layer 1, participants can tick the completed task.

Data quality checks and data cleaning

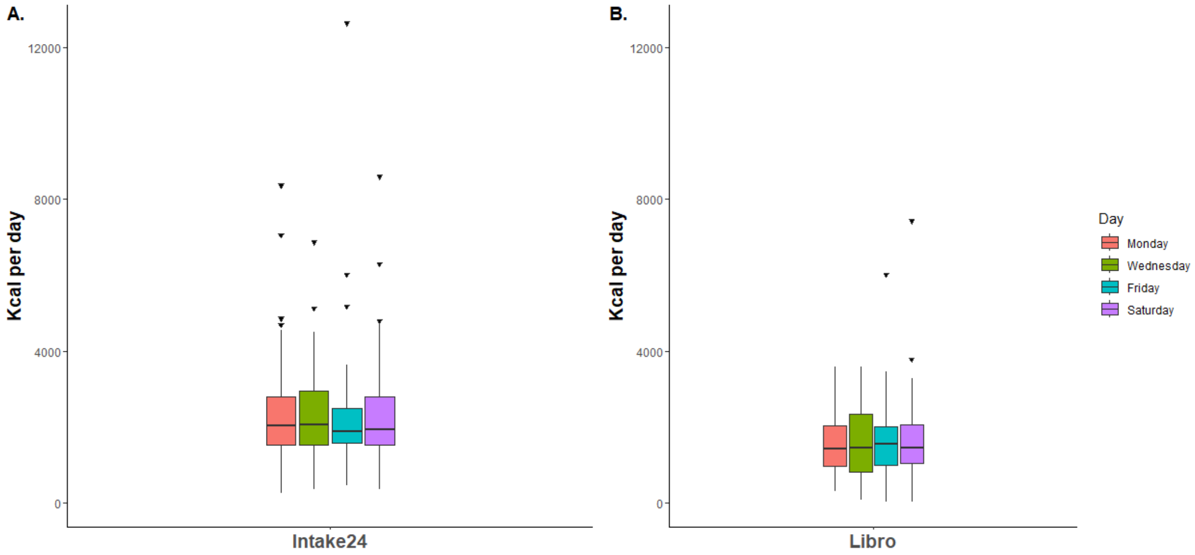

Figure S3. Daily single recalls of total Energy intake per each method. The distribution of daily single recalls of total energy intake by day from all participants recorded with Intake24 (left) and Libro (right) including 49 subjects. Daily EI captured by Intake24 showed a higher number of outliers with a trend towards the higher end. Manual inspection of the recorded items found a few implausible portion sizes underlying these records. Single recalls higher than 5000 Kcal were then removed for both methods from further analysis. Data quality was also inspected in terms of underreporting defined as EI < 400 Kcal. Three SRs (1.6%) were identified for Intake24, although no participant had an average EI below 400 Kcal when considering the mean of their recorded SRs. Ten SRs (5.3%) recorded using Libro were lower than 400 Kcal, with two participants (4.1%) reporting less than 400Kcal on average across all their SRs.

Individual and group agreement between methods

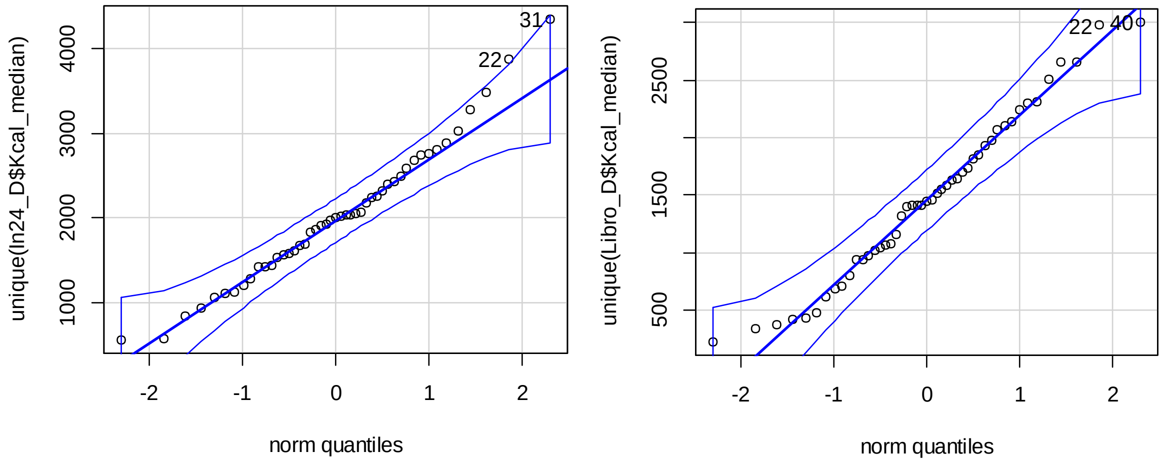

Figure S4. Qq plots for median EI. Qq plots for median EI from single recalls for each participant (left: Intake24, right: Libro).

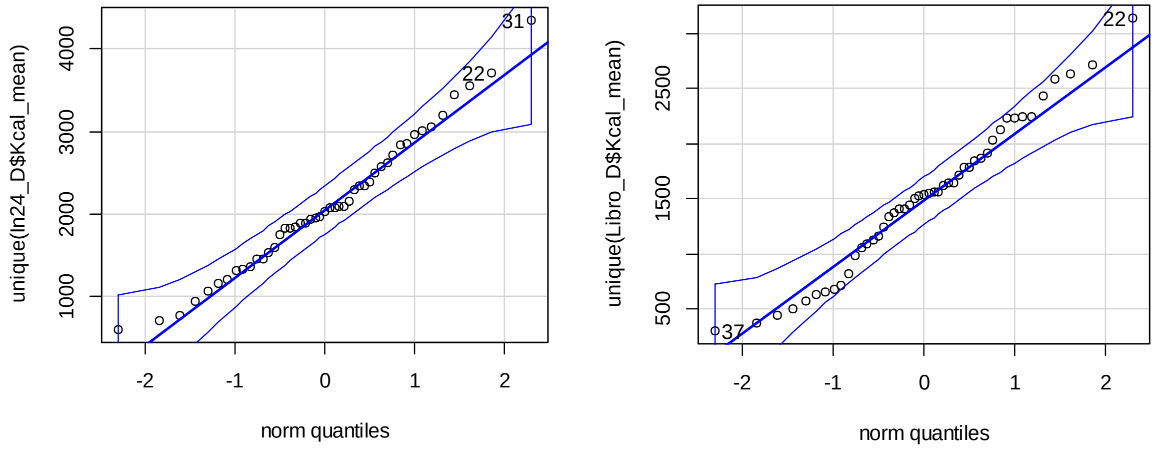

Figure S5. Qq plots for mean EI. Qq plots for mean EI from single recalls for each participant (left: Intake24, right: Libro).

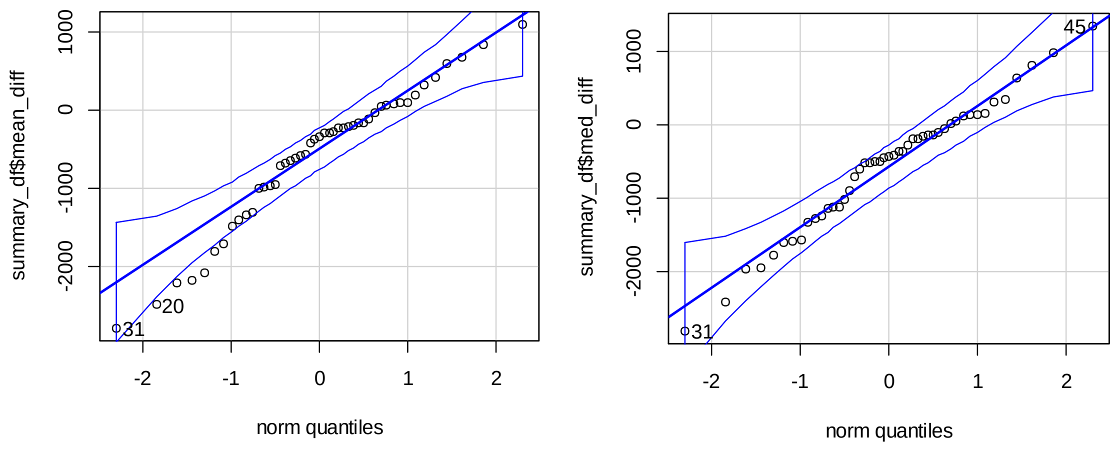

Figure S6. Qq plots for Libro – Intake24 differences. Qq plots for differences between Libro and Intake24 intake using mean (left) and median (right) values of single recalls for each participant.

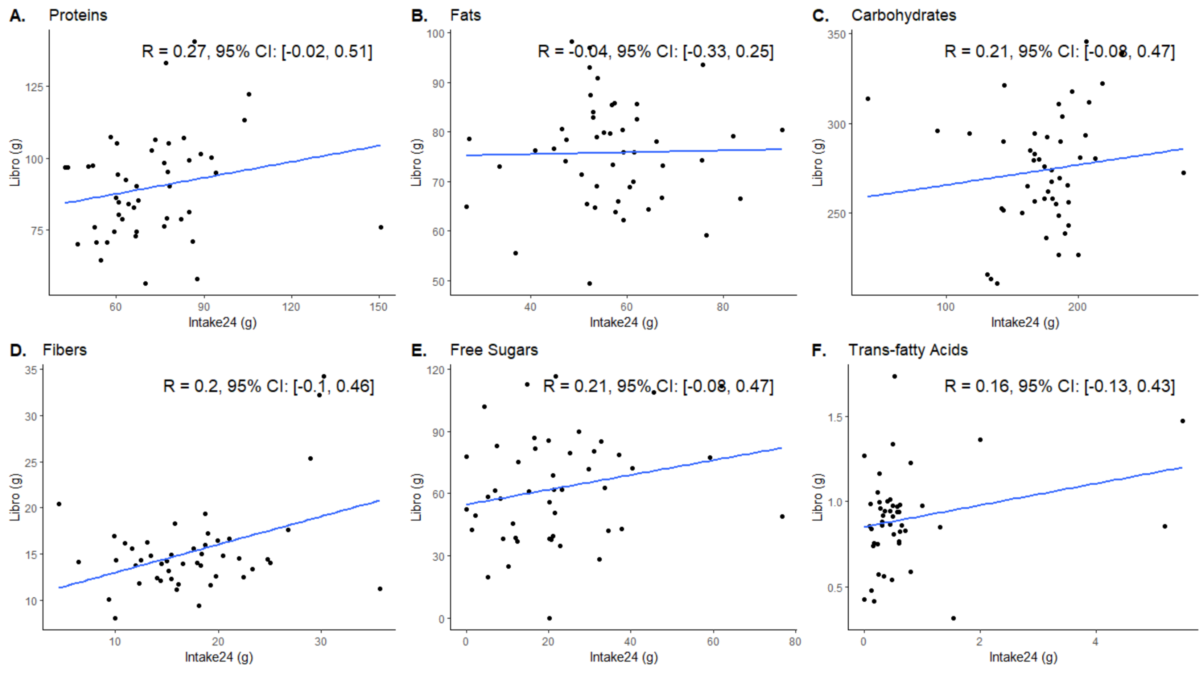

Figure S7. Scatter plot of nutrient intake. Scatter plot of Libro versus Intake24 mean values with a linear regression line for (A.) protein intake, (B.) fat intake, (C.) carbohydrates intake, (D.) fibre intake, (E.) free sugar intake, (F.) trans-fatty acids intake.
